# Plant microRNAs as novel immunomodulatory agents

**DOI:** 10.1038/srep25761

**Published:** 2016-05-11

**Authors:** Duccio Cavalieri, Lisa Rizzetto, Noemi Tocci, Damariz Rivero, Elisa Asquini, Azeddine Si-Ammour, Elena Bonechi, Clara Ballerini, Roberto Viola

**Affiliations:** 1 Research and Innovation Centre, Fondazione Edmund Mach, via E. Mach 1, 38010 San Michele all’Adige (TN), Italy; 2 Department of Biology, University of Florence, Via Madonna del Piano 6, 50019 Sesto Fiorentino (FI), Italy; 3 Dipartimento di Neuroscienze, Psicologia, Area del Farmaco e Salute del Bambino (Neurofarba), University of Florence, viale Pieraccini 6, 50139 Firenze, Italy

## Abstract

An increasing body of literature is addressing the immuno-modulating functions of miRNAs which include paracrine signaling via exosome-mediated intercellular miRNA. In view of the recent evidence of intake and bioavailability of dietary miRNAs in humans and animals we explored the immuno-modulating capacity of plant derived miRNAs. Here we show that transfection of synthetic miRNAs or native miRNA-enriched fractions obtained from a wide range of plant species and organs modifies dendritic cells ability to respond to inflammatory agents by limiting T cell proliferation and consequently dampening inflammation. This immuno-modulatory effect appears associated with binding of plant miRNA on TLR3 with ensuing impairment of TRIF signaling. Similarly, *in vivo*, plant small RNAs reduce the onset of severity of Experimental Autoimmune Encephalomyelities by limiting dendritic cell migration and dampening Th1 and Th17 responses in a Treg-independent manner. Our results indicate a potential for therapeutic use of plant miRNAs in the prevention of chronic-inflammation related diseases.

The discovery of microRNAs (miRNAs) and their interaction with the gene expression machinery in most organisms is one of the major scientific breakthroughs in recent years^1^. The regulatory effects of miRNAs have been mainly elucidated studying their expression within a given cell type^2^. Endogenous miRNAs are known to mediate gene expression of cognate messenger RNAs (mRNAs) in a sequence specific manner by affecting, via the RNA-induced silencing complex (RISC), fundamental processes ranging from cell development to cancer to immune regulation^3^,^4^,^5^,^6^,^7^. Yet, even still controversial, miRNAs have been shown also to act outside the cell from which they originate by long distance transport^8^,^9^,^10^,^11^,^12^,^13^.

The inter-cellular mode-of-action of miRNAs has led to suggestions for therapeutic applications^14^ in analogy with exogenously supplemented small interfering RNAs (siRNAs), which induce degradation of sequence-specific homologous mRNA via RNA interference. In the case of siRNAs, “off-targeting effects” and adverse reactions such as the promotion of inflammation^15^,^16^ have so far hampered their wider adoption as therapeutic application^17^. Exogenous double stranded RNA (dsRNA) as well as single stranded RNA (ssRNAs) are intercepted by the Pathogen Recognition Receptors (PRRs) of the innate immune system such as membrane-bound Toll-Like Receptors (TLRs) and cytoplasmic receptors abundant in the immune cell^18^,^19^ and activate an inflammatory response through the type I IFN system^20^. While TLR7 and TLR8 recognize ssRNAs^21^, TLR3 binds to both single stranded^22^ and double stranded viral RNAs^23^,^24^. TLR3 has also been shown to mediate siRNA non-sequence-specific immune suppression through the type I IFN system in cultured mammalian cells^20^. TLR7 and TLR8 also appear to be involved in siRNA pro-inflammatory effects^25^ and in miRNA-mediated paracrine loop between cancer cells and immune cells present in the tumor microenvironment with TLR-mediated pro-metastatic inflammatory response that ultimately may lead to tumor growth and metastasis^26^.

In addition to intercellular exchange within organisms^8^,^26^,^27^, inter-organismal miRNA exchanges are also known to occur including between taxonomic kingdoms as suggested by the recently reported presence of dietary plant microRNA in plasma and organs of humans and animals^10^,^28^,^29^,^30^. These latter findings raise paradigm-changing questions concerning our understanding of diet-health interactions. However, the validity of these claims has been challenged as some authors consider artefactual the detection of dietary plant miRNA sequences in the plasma after different feeding regimes^28^,^31^,^32^,^33^ while others do not question the presence of plant miRNAs in plasma or internal organs, but note that the reported copy number of individual sequences appears too low to be biologically relevant^28^,^34^. Plant-based dietary delivery of miRNA for therapeutic purposes has also been proposed^10^,^35^.

Since the immune function is the likely mediator of such interactions we have sought to explore the impact of plant miRNAs on dendritic cells (DCs), a component of the innate immune system present in the gut and responsible for instructing T cells to react and adapt to environmental challenges. Here we show that synthetic *Fragaria vesca* miR168 at physiological concentrations reduce inflammation mediated by TLR agonists via a TLR3-mediated mechanism. Indeed, this efficacy was not limited to miRNA from strawberry but was extended to sRNAs extracts from a wide variety of plant tissues and species. Furthermore, treatment with plant sRNAs could systemically reduce inflammation and prevent symptoms of multiple sclerosis in an Experimental Autoimmune Encephalomyelities (EAE) mouse model. These results provide a novel mechanistic explanation for known beneficiary effects exerted by fruit and vegetable in the prevention of inflammation associated disorders.

## Results

### Strawberry fruit FvmiR168 affects DC properties and their ability to respond to inflammatory stimuli by limiting T cell proliferation

**Figure 1:**
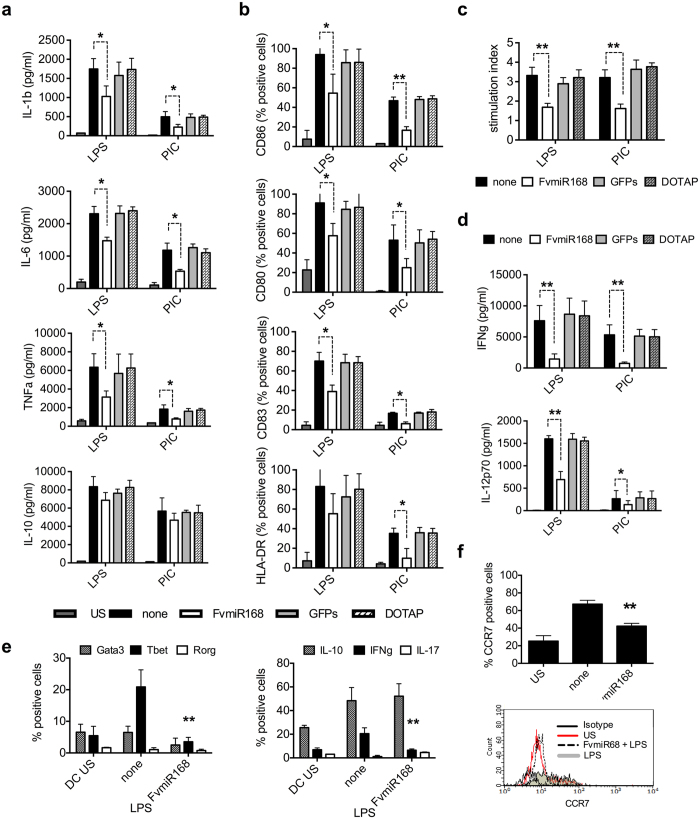
Effects of plant miRNA treatment in human immune function. (**a**,**b**) Effects of miRNA treatment on DC activation. DCs were exposed for 2 hr to *Fv*miR168 (10 ng/ml) and then exposed to LPS or PolyI:C (PIC, 10 ng/ml). After 24 hr IL-1β, TNFα, IL-10, IL-6 released on supernatant (**a**) and costimulatory markers’ expression on DC surface (**b**) have been measured. (**c**) Effects of treated DCs in stimulating T cells. T cells were exposed to DCs, in presence or not of miRNAs (10 ng/ml) and LPS or PolyI:C (10 ng/ml). After five days, proliferation has been measured as [^3^H]-Thy uptake by liquid scintillation [^3^H]-Thy. Data have been expressed as stimulation index: percentage of stimulation above background is determined for each stimulated sample, through comparison with results from an unstimulated sample. (**d**) Superntants have been collected, before addition of [^3^H]-Thy and INFγ release was assayed. In parallel, on the DCs used for the MLR assay IL-12p70 have been measured on 24 h supernatants. (**e**) In parallel, on the same set of T cells stimulated with LPS were not treated with [^3^H]-Thy and to characterize the T cell population present in the samples, intracellular staining was performed to evaluate the expression of Tbet, Rorγt and Gata3 by flow cytometry as well as the intracellular cytokine production for IL-10, IFNγ and IL-17. Data are presented as mean + SD, N = 5, *p < 0.05, **p < 0.01, Student t-test, pretreatment (+*Fv*miR168) *vs* no pretreatment (none, LPS or PIC alone). (**f**) CCR7 expression on DCs surface. DCs were exposed for 2 hr to miRNAs (10 ng/ml) and then exposed to LPS (10 ng/ml) for 24 hr. Thereafter, CCR7 expression was evaluated by flow cytometry on CD11c^+^ gated cells. Data are presented as mean + SD, N = 3, *p < 0.05, **p < 0.01, Student t-test, pretreatment (+*Fv*miR168) *vs* no pretreatment (none, LPS or PIC alone).

For initial experiments we tested the immuno-modulatory efficacy of *Fragaria vesca* 3′ end methylated miR168 (Table 1), one of the most abundant miRNAs present in strawberry fruits, an example of edible miRNA rich plant tissue that is consumed unprocessed. When human monocyte-derived DCs were treated with *Fv*miR168 complexed with DOTAP at miRNA concentrations up to 10 μg/ml,no effect was observed (Supplementary Fig. 1). However, when *Fv*mir168 pre-treated DCs were challenged with the inflammatory agents LPS or polyI:C, a significant reduction of their inflammatory response was observed (Fig. 1). *Fv*miR168 decreased LPS- and PolyI:C-induced production of IL-1β and TNFα (Fig. 1a, Student t-test, p < 0.05). *Fv*mir168 also reduced the levels of CD80, CD86, CD83 and the class II immuno-histocompatibility complex (HLA-DR) (Fig. 1b, p < 0.05). Significantly, these effects were observed at a concentration of 10 ng/mL, a level three order of magnitude lower than that required to induce a similar effect by human anti-inflammatory miRNAs^36^,^37^. *Fv*miR168 treated-DCs also showed a reduced ability to induce T cell proliferation in the presence of LPS or polyI:C (Fig. 1c, p < 0.01). The amount of IFNγ produced by T cell was also reduced (p < 0.01), in accordance with the reduced ability of DCs to secrete IL-12p70 (Fig. 1d, p < 0.01). Th1 cell differentiation was indeed impaired by the *Fv*miR168 pre-treatment of DCs as revealed by reduction of both Tbet and IFNγ expression (p < 0.01). On the other hand, as expected, Th2 differentiation was not affected (Fig. 1e). *Fv*miR168 treated-DCs also showed a significant reduction in the expression of CCR7 (Fig. 1f, p < 0.01), a molecule known to guide DCs to and within lymphoid organs^38^, suggesting a defect in their migration ability.

### FvmiR168 interacts with DCs through TLR3

**Figure 2:**
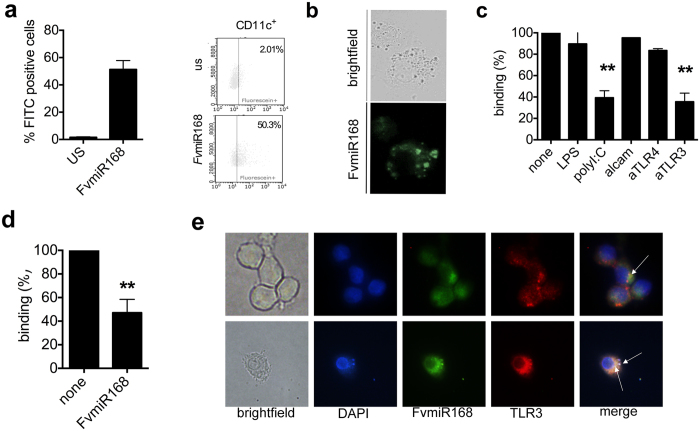
Plant miRNA interacts with TLR3. (**a**,**b**) DCs enter in contact with plant miRNA. DCs were treated for 2 hr with 10 ng/ml FITC-labeled *Fv*miR68. Contact has been evaluated by assessing the number of green cells by flow cytometry on specifically gated population (**a**). Data are presented as mean + SD, N = 3 plus representative plots. (**b**) Representative images of green fluorescent cells after *Fv*miR168 treatment. (**c**) DCs bind *Fv*miR168 through TLR3. CD11c-APC–labeled DCs were incubated with FITC-labeled *Fv*miR168 in the presence or absence of LPS, LMW poly:IC (PIC), anti–TLR4 mAb, anti-TLR3 mAb, and anti–ALCAM Ab as control. Cells were then analyzed for positivity to FITC by flow cytometry. Basal binding (TSA) is set as 100%. Data are presented as mean ± SD (N = 4). **p-value < 0.01, Student t-test, blocked *vs* not-blocked cells (none). (**d**) PolyI:C competes with miRNA for TLR3 binding. CD11c-APC–labeled DCs were incubated with FITC-labeled PolyI:C in the presence or absence of *Fv*miR168. Cells were then washed and analyzed for positivity to FITC by flow cytometry. Basal binding (TSA) is set as 100%. Data are presented as mean ± SD (N = 7). *p-value < 0.05, **p-value < 0.01, Student t-test, blocked *vs* not-blocked cells (none). (**e**) Fluorescence microscopy reveals that TLR3 (in red) localizes with *Fv*miR168 (green) in DCs (nucleus staining in blue, DAPI) as indicated by white arrows.

Extensive binding of *Fv*miR168 to DCs was observed when these cells were exposed to fluorescein-labeled *Fv*miR168 and analysed by flow cytometry (Fig. 2a) and fluorescence microscopy (Fig. 2b). *Fv*miR168 binding to DCs was reduced by pre-treatment with specific antibodies against TLR3 or LMW polyI:C (an TLR3 agonist) but not by pre-treatment with TLR4 antibodies or LPS (an TLR4 agonist) (Fig. 2c, Student t-test, p < 0.01). Conversely, pretreatment of DCs with *Fv*miR168 reduced polyI:C binding by almost 50% (Fig. 2d, p < 0.01). Fluorescence microscopy showed that FITC-labeled *Fv*miR168 distributes in the citoplasm forming clusters that co-localize with endosomal TLR3 (Fig. 2e).

### FvmiR168 interferes with TRIF signaling

**Figure 3:**
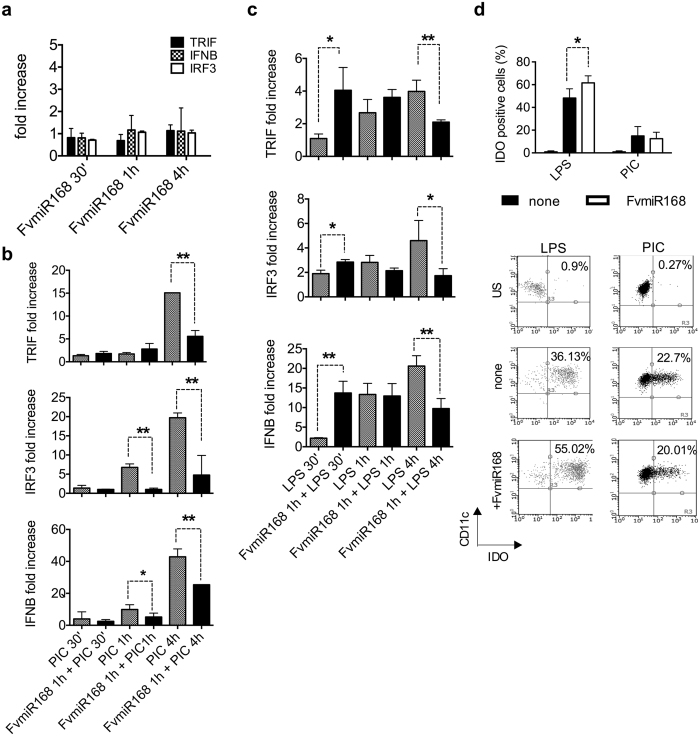
Plant miRNA acts on TRIF-mediated signaling. (**a**) DCs were treated or not for 30’ and 1 hr with 10 ng/ml *Fv*miR68. Cells were then collected, RNA extracted and levels of *TRIF* (TICAM1), *IFNB*, and *IRF3* were detected by quantitative real-time PCR. (**b**,**c**) DCs were treated or not for 1 hr with 10 ng/ml *Fv*miR68, and then exposed for 30’, 1 hr and 4 hr to LPS (b) or PolyI:C (b, PIC). Cells were then collected, RNA extracted and levels of *TRIF* (TICAM1), *IFNB*, and *IRF3* were detected by quantitative real-time PCR. Results are shown as means ± SD (N = 3). *p-value < 0.05, **p-value < 0.01, Student t-test treated (+*Fv*miR168) *vs* not-treated cells (none, LPS or PIC alone). (**d**) DCs were treated or not for 2 hr with 10 ng/ml FvmiR68 and then exposed for 20 hr to LPS (10 ng/ml) or PolyI:C (10 μg/ml). IDO expression has been analyzed by intracellular straining on gated CD11c^+^ DCs. Results are shown as means ± SD (N = 3). Above the graph a representative flow cytometry dot plot has been shown. *p-value < 0.05, Student t-test, pretreatment (+*Fv*miR168) *vs* not-pretreated cells (none).

We next examined the effect of *Fv*miR168 on the TRIF signaling pathway shared by TLR3 and TLR4. In DCs incubated with *Fv*miR168 for up to 1 hr no changes in the transcript levels of *TRIF*, *IFNB*, *IRF3* was observed (Fig. 3a). When *Fv*miR168 pre-treated DCs were subsequently incubated with polyI:C or LPS, a significant reduction of *TICAM1* (*TRIF*,) *IRF3* and *IFNB* mRNA was observed after 4 hr as compared with untreated DCs (Fig. 3b,c, Student t-test, p < 0.01). However, with LPS (but not polyI:C), the reduction of these transcripts was preceded by a significant but transient increase already after 30 minutes of treatment (Fig. 3c, p < 0.05), suggesting the involvement of a negative feedback mechanism. This hypothesis is reinforced by the observation that the percentage of IDO^+^ cells in the DC population in following 2 hr pre-treatment with Fvmir168 was significantly increased after 20 hr exposure to LPS but not PolyI:C (Fig. 3d, p < 0.05).

### The immuno-modulatory effect of plant miRNA is not sequence or plant specific

**Figure 4:**
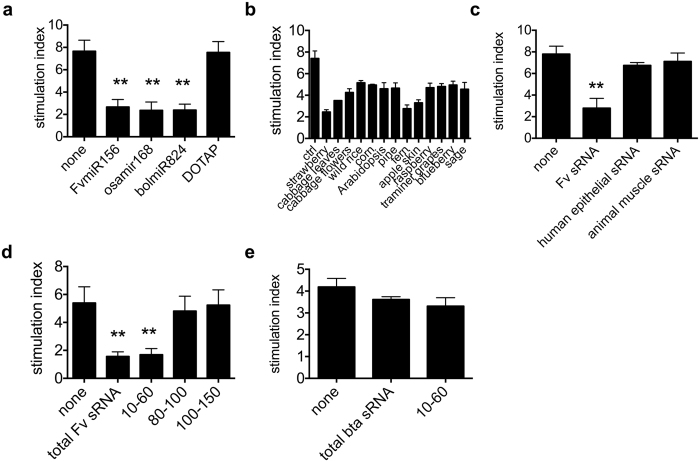
Effects of different plant sRNA extracts on T cell proliferation. (**a**–**d**) T cells were exposed to DCs, in presence or not of *Fv*miR146, *bol*miR874 or osamiR168 (10 ng/ml, (**a**), *F. vesca* total sRNA (**b**) or human and *B. taurus* total sRNA (10 ng/ml, (**c**) or sRNA fractions obtained as described in Material and Methods section (10 ng/ml, (**d**) or *B. taurus* total and 10–60 nt sRNA fraction (**e**), and LPS (10 ng/ml). Fractioning of sRNA has been performed by polyacrylamide electrophoresis separation. Panel (**d**) refers to the results obtained using 10–60 nt sRNA, 70–100 nt sRNA and 100–150 nt sRNA. After five days of T cells-DCs co-cultures, proliferation has been measured as [^3^H]-Thy uptake by liquid scintillation [^3^H]-Thy. MLR results are shown. Proliferation is presented as stimulation index, Percentage Stimulation above background is determined for each stimulated sample, through comparison with results from an unstimulated sample. Mean ± SD, N = 6, *p < 0.05, **p < 0.01, Kruskal Wallis, pretreatment (+miRNA duplexes or sRNA fractions) *vs* not-pretreated cells (none).

Given that dsRNA binding to TLR3 is not sequence-dependent, we tested whether the anti-inflammatory efficacy shown by *Fv*miR168 could also be observed with other miRNAs such as *Fv*miR156, another abundant strawberry miRNA, *bol*miR874 an abundant cabbage miRNA or *osa*miR168 from rice, the plant miRNA detected in blood plasma of human and animal subjects after dietary consumption of rice^29^. DCs treatment with synthetic equivalents of these plant miRNAs reduced T cell proliferation to a similar extent than *Fv*miR168 (Fig. 4a, p < 0.01).

To investigate the possibility that the observed effect may be a general property of plant miRNAs, we obtained RNA extracts from a range of plant species and organs including strawberry, blueberry, raspberry and grapevine fruits, cabbage, sage, pine and thale-cress leaves, fern fronds, apple peel, cabbage flowers, and wild rice and corn caryopses. The sRNA fraction of each extract was separated from the high molecular weight RNA and incubated at a concentration of 10 ng/ml with DCs. All of the tested extracts significantly attenuated the LPS-induced T cell proliferation with some extracts (sRNA from cabbage leaves, fern fronds, and apple peel) inducing an anti-inflammatory response equal or greater than *Fv*miR168 alone (Fig. 4b, p < 0.01). On the contrary, sRNA extracted from human epithelial (Caco2-derived) cells or beef muscle did not exert any anti-inflammatory effect (Fig. 4c). To evaluate the active fraction in the sRNA immuno-modulatory properties, plant-derived total sRNAs have been exposed to polyacrylamide gel fractioning as reported in Material and Methods. Among the three fractions recovered, the sRNA fraction comprised between 10 and 60 nt, enriched in 21nt sequences, including miRNAs (Supplementary Fig. 2), showed a strong anti-inflammatory activity (p < 0.01) whilst, the fractions from 70 up to 150 nt did not have any significant effect (Fig. 4d). Conversely, the 10–60 nt fraction from total sRNA extracted from beef muscle did not exert any anti-proliferative effect on T cells (Fig. 4e).

### 3′-OH methylation partially accounts for plant miRNA immuno-modulatory property

**Figure 5:**
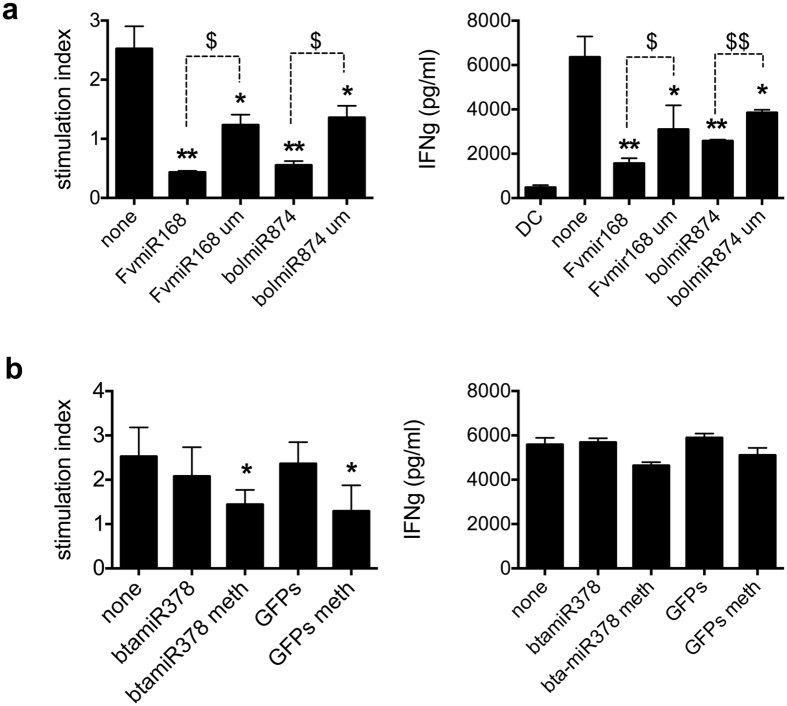
Effect of plant miRNA 3′OH-methylation on T cell proliferation. (**a**) T cells were exposed to DCs, in presence or not of 10 ng/ml methylated *Fv*miR168, *bol*miR874 or their un-methylated analogs. After five days of T cells-DCs co-cultures, proliferation has been measured as [^3^H]-Thy uptake by liquid scintillation. MLR results are shown. Proliferation is presented as stimulation index, Percentage Stimulation above background is determined for each stimulated sample, through comparison with results from an unstimulated sample. In parallel, IFNγ was assayed on 5-days supernatants collected before [^3^H]-Thy addition. (**b**) Similarly, T cells were exposed to DCs, in presence or not of 10 ng/ml *bta*miR378 or the GFPs duplex and their methylated analog. MLR results and IFNγ production are shown. Mean ± SD, N = 4, *p < 0.05, **p < 0.01, Kruskal Wallis, pretreatment (+miRNA duplexes or sRNA fractions) *vs* not-pretreated cells (none). ^$^p < 0.05, ^$$^p < 0.01, Student t-test, methylated *vs* un-methylated miRNA pretreatment. um, un-methylated; meth, methylated.

As plant miRNAs are characterized by a 2′-OH-methylation at their 3′ end^39^, we next explored whether this structural modification may explain their sequence-independent mode of action. Treatment with un-methylated forms of *Fv*miR168 or *bol*miR874 significantly reduced LPS-induced T cell proliferation and IFNγ production, albeit to a lower extent than treatment with the methylated forms (Fig. 5a). On the other hand, an anti-inflammatory efficacy, albeit statistically less significant than that exerted by plant miRNAs, could be conferred to miR378 from beef muscle (*bta*miR378) or GFPs duplex by introducing a methylation on the 3′ end (Fig. 5b).

### Plant sRNAs reduce the pathology development of EAE in mice

We next explored the efficacy of plant sRNA extracts in reducing the inflammatory process *in vivo* using a mouse model of multiple sclerosis (Experimental Autoimmune Encephalomyelitis, EAE)^40^ induced in C57BL/6 mice by immunization with myelin oligodendrocyte glycoprotein peptide 35–55 (MOG_35–55_ peptide). We tested a “cocktail” containing equal parts of the most effective sRNA (extracted from cabbage leaves, fern fronds and apple peel) dissolved in PBS and DOTAP (vehicle) to give the final dose of each extract of 10 μg/ml. sRNA cocktail or vehicle were given by intravenous injections at the early stage of the disease from three days post immunization (d.p.i.) and every four days for the entire duration of the disease (up to 25 d.p.i.) and the effects on EAE severity examined daily.

**Figure 6:**
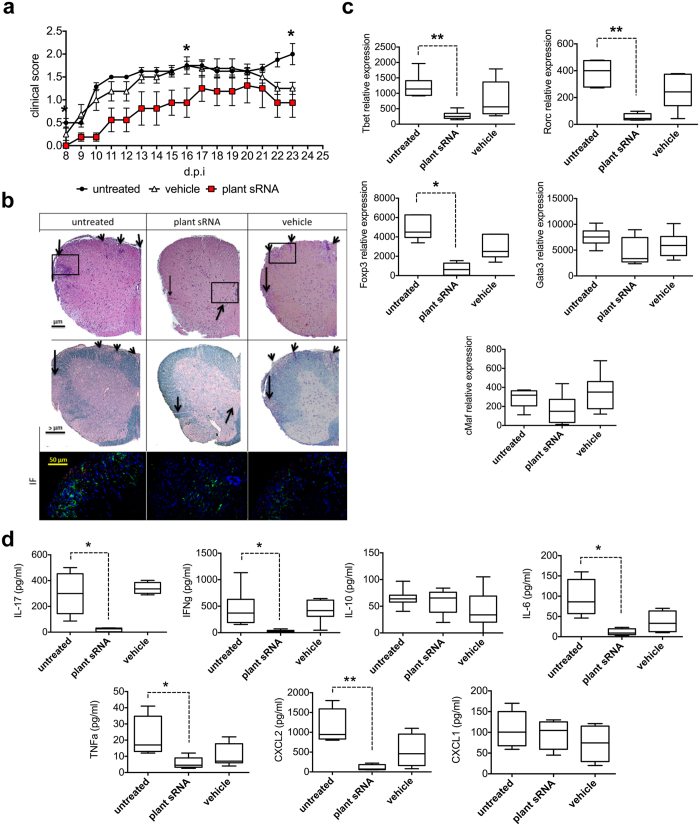
Effect of plant sRNA in EAE. (**a**) sRNA significantly ameliorated the clinical signs and disease progression of EAE. Analysis was performed by one way Anova test indicating that curves are statistically different (*p < 0.05). In particular clinical score is statistically significant in pool sRNA vs untreated mice and in pool sRNA vs vehicle treated mice at 8 and 15 days after immunization (d.p.i). (**b**) Effect of treatment with plant extract on inflammation and demyelination in the spinal cord of EAE mice. Sections were stained for Haematoxylin and Eosin (H&E) and LFB and examined to detect inflammatory infiltrates and demyelination, respectively, as indicated by arrows. Staining displayed a decrease of the number of infiltrates (arrows) in the white matter in the spinal cord of plant sRNA-treated mice compared to untreated or vehicle treated mice. Luxol Fast Blue (LFB) staining indicated a similar pattern after pool-sRNA treatment (arrows). Immunofluorescence analysis (IF) has also been performed. Section were stained with CD11c^+^ (RED) and Iba1 (green) to detect DCs and microglia respectively, and counterstained with DAPI (blue). Magnification of the black frames in upper panels are shown. CD11c infiltrates are absence in the spinal cord areas of immune cell invasion of plant sRNA treated mice. (**c**,**d**) *Ex vivo* analysis of T lymphocyte isolated from EAE lymphnodes. T cells were treated with *ex viv*o with MOG. To characterize the T cell population present in the samples, intracellular staining was performed to evaluate the expression of *Tbet*, *Rorc*, *Gata3*, *Foxp3* and *cMaf*. Mean ± SD of two independent experiments (**c**). On cytokine supernatans, IL-10, IL-17, IFNγ, TNFα, IL-6, CXCL1 and CXCL2 were measured (**d**). *p < 0.05, **p < 0.01, Student t-test, treatment *vs* no treatment.

No significant difference in body weight was observed between the treated groups and the untreated (control) group during the experimental period (data not shown). Mice from all groups developed the disease with the typical clinical signs. However, the plant sRNA-treated group displayed significantly attenuated clinical manifestation of EAE when compared to untreated and vehicle-treated mice (Fig. 6a). The mean of disease onset (8 d.p.i.) and maximum EAE score (15 d.p.i.) from the plant sRNA group was significantly lower compared to control groups (one way Anova, p < 0.05). These results indicated that plant extract administration conferred protection against EAE, promoting a less severe disease.

Following EAE induction, both untreated and plant sRNA- or vehicle-treated mice showed distinct areas of immune cell infiltration in the spinal cord but the plant sRNA-treated mice had visually less infiltrates than untreated or vehicle-treated mice (Fig. 6b). In addition, more numerous and larger plaques of demyelination were observed at the site of inflammatory cell infiltrates of untreated and vehicle-treated mice than in plant sRNA-treated mice (Fig. 6b). Hystological data correlate with immunohistochemical findings, where we show the absence of CD11c infiltrates in spinal cord areas of immune cell invasion (Fig. 6b, IF panel). These results indicate that the less severe disease developed by plant sRNA-treated mice is associated with improved histological outcomes in the spinal cord, due to difference in infiltrate composition. At the end of the experiment (25 d.p.i) *ex vivo* analyses of DC and T lymphocytes isolated from EAE lymphonodes of the three mice groups and measurements of cytokine production were performed. Analyses of the RNA levels of key transcription factors driving T-cell differentiation showed a reduction in the expression of master regulators of pro-inflammatory *Tbet* and *Rorc* (Student t-test, p < 0.01) genes but, unexpectedly, a significant reduction of the IL-10 master regulator *FoxP3* was also observed (Fig. 6c, p < 0.05). A significant reduction of the inflammatory cytokines IFNγ, IL-17, TNFα, CXCL2 and IL-6 in the sRNA-treated group was observed (Fig. 6d, Student t-test, p < 0.05, p < 0.01). Surprisingly, this reduction was not associated to an increase in the anti-inflammatory IL-10 production. These results indicate that the sRNA effect on the EAE inflammatory phenotype occurs via a FoxP3-independent mechanism.

Although CD11c^+^ DCs isolated from draining lymphonodes showed similar levels of MHCII, CD80, CD83 and CD86 expression in all treatments (Supplementary Fig. 3), both *in vivo* and *in vitro* suggesting a reduced efficiency in T cells polarization toward encephalytogenic Th1 and Th17 production and reduced CNS migratory ability.

## Discussion

DCs are the master regulators of immune signaling, orchestrating responses to external signals and instructing T cells in the type of inflammatory or tolerogenic responses to be exerted, sensing both live microbial cells and other microbial antigens, including RNAs^41^. Over the past decade a substantial amount of scientific literature has highlighted the fundamental regulatory role played by miRNAs in the regulation of DC function^7^,^37^,^42^, and development, differentiation, and function of T and B cells in autoimmune diseases^43^. miRNAs can also be naturally transferred via exosomes^9^,^27^,^44^ and miRNA-mediated cross-talk between cancer cells and neighboring immune cells with ensuing pro-inflammatory changes through direct binding to PRRs was demonstrated^26^,^45^. Thus, the recent evidence presented in support of plant miRNA uptake through the diet in human and animals, poses intriguing questions concerning the role of the immune system in diet-health interactions. We report here that exposure of DCs to synthetic strawberry fruits *Fv*miR168, packaged in exosome-like structures, does not modify their ability to induce T cell proliferation and differentiation. However, subsequent exposure of *Fv*miR168-treated DCs to LPS or polyI:C, agonists of TLR4 and TLR3 respectively, results in a strong reduction in the inflammatory response and this effect appeared associated to a decreased expression of TRIF transcript, downstream transducer of TLR3 and TLR4^46^. *Fv*miR168 appears to interact directly with TLR3 as demonstrated by its intracellular co-localization with endosomal TLR3 and decreased binding in the presence of TLR3 antibody. This finding is not surprising since TLR3 is the transmembrane PRR deputed to detection of dsRNA^23^. Although dsRNA ligands must be at least 45–49 nt in length to bind to TLR3 ectodomains and induce its functionalization through cooperative dimerization^47^,^48^, activation of TLR3 signaling has also been observed with shorter (from 20 to 40 nt) dsRNA ligands such as siRNAs^49^,^50^.

However, here we show that binding of *Fv*miR168 (21 nt in length) to TLR3 in DCs not only does not activate their pro-inflammatory response but it strongly represses their signaling in the presence of polyI:C and, surprisingly, LPS. Our results are consistent with the effects induced by dsRNA species below 20 nt in length which were shown to bind to the ectodomain of TLR3^51^ but failed to induce stable homodimer complexes^52^ and ensuing activation of the downstream signaling^47^,^49^. One possibility is that *Fv*miR168 pre-treatment of DCs promotes unproductive sequestration of TRIF adaptor to the TLR3 TIR domain, resulting in down-regulation of TRIF signaling following DCs exposure not only to polyI:C but also LPS, a TLR4 agonist. Intriguingly, the dynamics of TRIF signaling transcripts following LPS treatment indicate the induction of a feedback loop involving IDO expression which is known to be TRIF dependent^53^ and IDO-competent DCs are capable of inhibiting T cell proliferation *in vitro*^54^ and might induce tolerance *in vivo*^55^.

Although a direct impact of *Fv*miR168 on TRIF mRNA cannot be excluded, the finding that anti-inflammatory efficacy of *Fv*miR168 could be replicated by other plant miRNAs sequences (*Fv*miR156, *bol*miR874, *osa*miR168) and even miRNA-rich sRNA extracts from a wide range of plants, suggests the involvement of a sequence-independent mechanism, such as interaction with PRRs. Indeed, this property appears to be a sequence-independent “class-attribute” of plant miRNAs as it was also observed with the fraction of 10–60 nt in length of sRNA extracts obtained from a wide range of plant species but not from mammalian sources (human Caco2 cell line or *Bos taurus* muscle). As nucleosides methylation of dsRNA interferes with their ability to initiate sequence-independent pro-inflammatory response^56^ and 2′-O-methylation at the 3′ end is a class attribute of plant miRNAs and siRNAs^39^, we reasoned that such structural modification may be responsible for the specific properties of plant sRNA extracts. However, removal of the 3′ end methylation from plant miRNAs sequences (duplexes) reduces but does not abolish their anti-inflammatory potency. Moreover, the introduction of a 2′-O-methylation at the 3′ end of mammalian or GFP miRNA duplexes, although effective in conferring some anti-inflammatory efficacy, it does not replicate that of methylated plant miRNAs. These results suggest that a 3′ end methylation of miRNAs duplexes is an important but not sufficient requisite to explain their anti-inflammatory efficacy in our experimental system. It is feasible that 3′ end methylation of miRNA could promote their stability and/or directly mediate their interaction with TLR3. However, other structural properties are required to promote a full anti-inflammatory effect and these seem to be found specifically in natural plant miRNAs. Further work is required to unveil the exact mechanism underpinning the secondary plant miRNA structure responsible for their immuno-modulatory effects.

The therapeutic potential of plant miRNAs was confirmed in a mouse model of human multiple sclerosis, EAE induced by active MOG immunization, where intravenous injections of 1.5 μg of DOTAP-complexed plant sRNA extracts significantly attenuate the pathology development of EAE. Clinical amelioration was associated with decreased spinal cord inflammatory infiltrates, that vary also in cell composition, and consequent decreased demyelination. Treated animals showed different T cell subpopulation profiles with diminished IFNγ and IL-17 production, indicative of diminished lymphocytes polarization toward Th1 and Th17, master effectors of EAE. This shift was confirmed by the quantitative evaluation of T cell transcription factors that characterize the T cell phenotype. Although the sRNA treatment down-modulated FoxP3 expression, a transcription factor that normally identifies Treg, the expected reduction of IL-10 production was not observed. We know that FoxP3 may vary with cell activation^57^ increasing the relative expression in early-activated cells without affecting Treg differentiation. The reduced FoxP3 expression we observed in our sample is an indication of reduction in T cell activation following plant sRNA treatment. As our *in vitro* data suggest that this is not the result of a direct effect of plant miRNA on T cells, we may explain the *in vivo* results as DCs mediated action on immunogenic, MOG specific T cells. CD11c^+^ DCs from draining lymphonodes were apparently matured. Yet the plant sRNA-treated mice lymphonode isolated DCs may be less efficient in T cells polarization toward encephalytogenic Th1 and Th17 production, because of the reduced production of soluble factors, i.e. IL-12, IL-6 and TNFα. Furthermore, the reduced expression of CCR7 observed *in vitro* leads to impaired migratory ability of DCs in the presence of *Fv*miR168, and may explain the reduced efficiency of T cell priming and activation in lymphonodes and CNS observed *in vivo*. Altogether these results suggest that the plant sRNA effect on the EAE occurs via reduction of primary inflammatory signaling mediated by DCs, and consequently reduced priming of Th1 and Th17 cells formation at the immune synapse. This mechanism does not require FoxP3-mediated Treg differentiation and secondary suppression of inflammation. This evidence is in agreement with previous observations that TLR3 defective mice show reduced CNV inflammation^58^, indicating that interfering with Trif-TLR3 mediated inflammation is a viable approach for treatment of autoimmune disorders.

Thus we show here that the anti-inflammatory properties of plant miRNAs and sRNAs may have both preventive and therapeutic efficacies. By shaping the regulation of inflammation, dietary plant miRNAs could early on moderate responses to inflammatory stimuli later in life, thus reducing chronic diseases^59^. We conclude that better understanding of the cross talk between exogenous miRNA and immune function will open novel opportunities to the development of an entirely new class of miRNA based anti-inflammatory drugs.

## Methods

### Ethics statement

The study was designed in conformity with the international recommendation (Dir. EU 2001/20/EC) and its Italian counterpart (DM 15 Luglio 1997; D.Lvo 211/2003; D.L.vo 200/2007) for clinical trial and following the Declaration of Helsinki, to assure protection and care of subjects involved. Human peripheral blood mononuclear cells (PBMCs) were isolated from buffy coats provided by Careggi Hospital (Florence, ethical approval document n. 87/10), or by Centro Trasfusionale of Santa Chiara Hospital (Trento, ethical approval document n. 54896583) after obtaining ethical approval by their respective internal ethical committees, as indicated in brackets. Written informed consent has been obtained from all the adult subjects. All animal manipulations were performed in the animal facility at the CeSaL (University of Florence) according to the European Community guidelines for animal care (DL 116/92, application of the European Communities Council Directive 86/609/EEC) and the Guiding Principles for Research Involving Animals Beings. Experimental procedures were approved by the Committee for Animal Care and Experimental Use of the University of Florence.

### Synthetic miRNA

Synthetic miRNA duplexes (Table 1) were synthesized by Sigma Genosys and purified by HPLC with the addition of the specific methyl modification at the 3′ termini. The annealing of small RNA strands was performed as described by Sigma oligo synthesis service. Upon arrival the small RNA duplexes were resuspended in RNAse-free water to the appropriate concentration. In each experiment miRNAs were complexed with DOTAP Liposomal Transfection Reagent (Roche) which facilitates nucleic acid entry into the cells^36^,^60^. In each experiment, DOTAP is also added to the control cells to exclude cross reactivity.

### Cell preparation

The PBMC fraction was obtained by density centrifugation of diluted blood over Ficoll-Paque (GE Healthcare). Monocytes were isolated from low density PBMCs by magnetic enrichment with anti-CD14 MicroBreads (Miltenyi Biotec) and cultured as previously described to allow DC differentiation^61^. DC activation was induced by LPS (10 ng/ml, Sigma Aldrich) or Low Molecular Weight (LMW) PolyI:C (10 μg/ml, InvivoGen). Total CD4^+^ T cells were obtained from PBMC by negative isolation with a combination of magnetic sorting (CD4^+^ T Cell Isolation II Kit). Depending on the experiment, supernatants were collected after 24 hr or 5 d, and stored at 20 °C until the assays. Cells viability was assessed before each experiment by Trypan blue (Euroclone) exclusion. DC viability was also evaluated after each experiment by flow cytometry using the Annexin V-FITC kit (BD Pharmingen) (Supplementary Fig. 4). The viability was always higher than 95% in all the experiments performed.

### Mixed lymphocyte reaction (MLR)

1 × 10^5^ CD4^+^ T cells were incubated, in 96-well U bottom plates, for 5 d in RPMI with 10% FBS together with allogeneic DCs, in presence or not of miRNA (10 ng/ml) and LPS or PolyI:C. At day 5, the proliferative response was measured by the [^3^H]-thymidine ([^3^H]-Thy, 1 μCi/mL, Amersham Bioscience) incorporation test. [^3^H]-Thy was added for the last 8 hr of incubation. Plates were harvested (Tomtec Mach III, Wallac) on glass fiber filters (PerkinElmer), and [^3^H]-Thy uptake was measured by liquid scintillation in a Microbeta 1450 Trimux counter (Wallac). The proliferative response is reported as a stimulation index (SI, mean cpm response/mean cpm background).

### Human cytokine assay

At the indicated times, supernatants from human cell cultures were collected and cytokine detection was performed using the Milliplex^®^ MAP human cytokine/chemokine kit (Merck-Millipore), according to the manufacturer’s instructions. IL-6, TNFα, IL-1β, IL-10, IL-12p70 and IFNγ were assayed.

### Human flow cytometry

For DC surface markers evaluation, cells were labeled with adequate concentrations of labeled antibodies in PBS with 1% FBS for 20 min at 4 °C, washed twice, and analyzed immediately. T cell intracellular staining for Tbet, Rorγt, IL-17A, IL-10 and IFNγ were performed using the fixation/permeabilization buffer kit (Life Technologies) following the manufacturing recommendations. A minimum of 10000 events for each sample were acquired using a Guava easyCyte 8T flow cytometer (inCyte software,Merck-Millipore). The area of positivity was determined by using an isotype-matched control MAb. Antibodies used: Fluorescein isothiocyanate (FITC)–anti-IL17A, phycoerythrin (PE)–IL10, APC–anti-CD4, APC-anti-CD11c, PE-IL-10, FITC-Tbet (Merck-Millipore), PE-Cy7 Tbet, APC-Rorγt, PE-IFNγ, PE-CD11c, (BD Biosciences Pharmingen); FITC-CCR7 (eBioscience); FITC-CD80, PE-CD86, ECD-HLADR and PC5-CD83 (Beckman Coulter).

### Binding assay

*Fv*miR168 was labeled with fluorescein using the 5′ End-Tag kit (Vendor Laboratories) following the manufacturer’s instruction. The labeled miRNA was then added at a concentration of 10 ng/ml to DCs. After 2 hr, cells were visualized for green-positivity by flow cytometry. In parallel, cells were visualized at fluorescence microscope. To test the effects of various reagents on ligand binding, the following concentrations were used: LPS (1 μg/ml), LMW poly:IC (10 μg/ml), anti–TLR4 mAb, anti-TLR3 mAb (both from InvivoGen) and anti–ALCAM Ab (30 μg/ml). Incubation was performed in 20 mM Tris, pH 8.0, 150 mM NaCl, 1 mM CaCl_2_, 2 mM MgCl_2_, and 1% BSA. After 1 hr of incubation at 37 °C, cell–miRNA conjugates were analyzed by flow cytometry. DCs were labeled with anti-CD11c-APC to discriminate cells binding FITC-labeled miRNA from free miRNA-containing vesicles.

### Quantitative real time PCR

Total RNA was extracted with the RNeasy Mini Kit (Qiagen, Valencia, CA). SuperScript^®^ VILO cDNA Synthesis Kit (ThermoFisher Scientific) was used for cDNA synthesis. Transcripts for *TRIF/TICAM*, *IRF3* and *IFNB1* were quantified with Applied Biosystems predesigned TaqMan Gene Expression Assays and reagents according to the manufacturer’s instructions. Quantification of the PCR signals was performed by comparing the cycle threshold value of the gene of interest with the cycle threshold value of the reference gene *GAPDH*. Values are expressed as fold increase of mRNA relative to that in not treated cells.

### smallRNA preparation

Plant, epithelial (Caco2-derived) and beef derived-sRNAs were extracted with the cetyltrimethylammonium bromide (CTAB)-based method^62^ or using the mirPremier microRNA Isolation Kit (Sigma-Aldrich) according to the manufacturer’s protocol. The use of CTAB together with Polyvinylpyrrolidone (PVP 40) followed by three successive chlorophorm:isoamyl alcohol extractions - or, as in the case of the kit, the use of filtration columns - allowed to denature and eliminate the protein complex present in the sample. After each extraction, samples will be analyzed using the Agilent RNA 6000 Nano Kit through the BioAnalyzer instrument (Agilent Technologies), for checking purity from high molecular weight RNA. Furthermore each sRNA preparation were analyzed using the Agilent Small RNA kit, to characterized the size of the sRNA present in each fraction. Size determination has been assessed on the basis of the retention time with respect to the known sizes and timing of the ladder peaks. The sRNA content ranged between 10 and 150 nt for all the plant used.

### Gel fractioning of sRNA

A standard 12% acrylamide gel [1X TBE, 12% acrylamide (19:1 acryl:bis-acryl)] was used to separate the plant sRNA. The gel was warmed to ~50 °C by running it for 10–15 minutes at 40 V in 1X TBE running buffer. 1 μg of sRNA was combined with an equal volume of Gel Loading Buffer II and then used for loading the gel. The gel was run until the leading dye travels about 4–5 cm down the gel, at 70 V. Gel was stained with SYBR Gold (Life Technologies) according to manufacturer’s extraction. Different gel slices (covering the range from 10 to 60 nt, 70–100 nt and 100–150 nt), were recovered according to the band profile and the two ladders used (the Low Range ssRNA Ladder and the microRNA Marker, both from New England BioLabs inc.) and crushed in 1 M NaCl and incubated overnight at 4 °C. The day after, the samples were centrifuged for 5 min at 2000 g and the supernatant transferred to a 50 ml tube. Crushed gel slices were re-elute with 2 ml of 1 M NaCl for 1 hr at room temperature and centrifuged again. The pooled supernatants were purified with MEGAclear Kit (Life Technologies). Obtained fractions were analyzed using the Agilent Small RNA kit, to check the sizing and purity of the fractionation performed as well as the miRNA (21 nucleotides) content, according to the manufacturer’s software.

### Induction and clinical assessment of EAE

Six to eight-week old C57Bl/6 female mice were obtained from Harlan, Italy Srl. The mice were housed in macrocolon cages on a 12 hr light/dark cycle at 23 °C, with ad libitum access to food and water. Adequate measures were taken to minimize pain and discomfort. Mice were immunized subcutaneously in the flanks and at the base of the tail with a total of 200 μg of MOG_35–55_ (synthesized by EspiKem Srl, Università di Firenze) per animal emulsified in complete Freund adjuvant (Sigma-Aldrich) supplemented with 4 mg/ml of *Mycobacteriun tibercolosis* (strain H37Ra; Difco Laboratories). Immediately thereafter, and 48 hr later, the mice received an intraperitoneal injection of 500 ng pertussis toxin (Sigma-Aldrich) in 100 μl of phosphate buffer saline (PBS). The animal were examined daily for weight loss and disability, and were clinically graded by investigators blind to group identity, as follows: zero indicate no signs, 0.5 partial loss of tail tonicity, 1 paralyzed tail, 2 ataxia and difficulty in righting, 3 paralysis of the hind limbs and/or paresis of the forelimbs, 4 tetraparalysis, 5 moribund or death.

#### Treatments

Plant sRNA (pool of three plants, total of 30 μg/mice), obtained as described above was freshly dissolved in PBS/DOTAP (60 μl/mice, stock solution 1 μg/μl before injection heated 20′ at 37 °C. 150 μl of PBS/DOTAP/plant extract or vehicle were intravenously injected every four days from 3 d.p.i. to 22 d.p.i. for a total of six injections. At 25 d.p.i. animals were sacrificed.

#### Neuropathological evaluations

At the time of sacrifice, the mice were anesthetized with pentobarbital (65 mg/kg, intraperitoneally). The spinal cord was removed from the column and fixed in 4% (v/v9 paraformaldehyde in PBS) and subsequently paraffin-embedded. Five micrometers thick transversal sections were cut and placed on glass slides. Serial sections were stained with Haematoxylin and Eosin (H&E), Luxol Fast Blue (LFB)-cresyl violet. Immunohistochemistry analysis was performed as reported^57^ with anti-Iba1 (rabbit anti-mouse, Wako Chemicals) and anti-CD11c (hamster anti-mouse , eBioscience) as primary antibodies and anti-rabbit AlexaFluor 488 and anti-hamster AlexaFluor 546 (eBioscience) as secondary antibodies.

#### Immunological evaluations

Cells were isolated from draining lymphonodes of EAE animals and analyzed for T cell proliferative response, dendritic cell phenotype, T cell cytokines production and expression of transcription factors. Briefly, DCs phenotypes were investigated for surface marker expression by mean of flow cytometry: after staining with fluorescent monoclonal antibodies directed against MHC II, CD11c, CD80, CD86 and CD83 (all eBioscience), cells were analyzed on a four-color Epics XL cytometer (Expo32 software; Beckman Coulter). T lymphocytes of MOG_35–55_ immunized mice treated or not were cultured in complete RPMI in 96 well plates (200000 cells/well) and stimulated with antigen. At day 3, the proliferative response was measured by ^3^H-thymidine incorporation test. Cytokines (IFNγ, IL1-7, TNFα, CXCL1, IL-6, CXCL2 and IL-10) were determined by Luminex (Kit Milliplex, Merck-Millipore) in cell supernatants harvested after 4 days of antigen stimulation (1 million cells/ml/well). Total RNA was extracted from lymphonodes’ cells isolated at sacrifice by using Qiazol following manufacturer’s protocol (Qiagen). cDNA was synthesized with RT quantitect (Qiagen). Real-time PCR was performed with an ABI Prism 7900HT Sequence Detection System (Applied Biosystems), according to manufacturer’s instructions. All PCR amplifications were performed with TaqMan Universal Master Mix and with Assay-on-demand (Applied Biosystems). Relative expression of mRNA levels was determined by comparing experimental levels with a standard curve generated with serial dilution of cDNA obtained from human PBMCs. Ubiquitin carboxy-terminal hydrolase L1 (*Hprt1* Hypoxanthine phosphoribosyltransferase 1) was used as a housekeeping gene for normalization. In each sample the level of the following mRNA level were evaluated: *Gata3*, *Tbet*, *Foxp3*, *cMaf* and *Rorc*. Ratio of transcription factors were calculated within each animal and means were compared.

## Additional Information

**How to cite this article**: Cavalieri, D. *et al*. Plant microRNAs as novel immunomodulatory agents. *Sci. Rep*. **6**, 25761; doi: 10.1038/srep25761 (2016).

## Supplementary Material

Supplementary Information

## Figures and Tables

**Table 1:** Sequences of the plant-derived miRNAs used.

*miRNA duplex*	*Sequence*(*5*′-*3*′)
FvmiR156	FvmiR156 UUGACAGAAGAGAGUGAGCAC
	FvmiR156* AGCUCUUUCUCUUUCUGUCACU
FvmiR168	FvmiR168 UCGCUUGGUGCAGGUCGGGAA
	FvmiR168* CCCGCCUUGCAUCAACUGAAU
osamiR168	osamiR168 UCGCUUGGUGCAGAUCGGGAC
	osamiR168* GAUCCCGCCUUGCACCAAGUGAAU
bolmiR824	bolmiR824 UAGACCAUUUGUGAGAAGGGA
	bolmiR824* CCTTCTCATCGATGGTCTAGA
btamiR378	btamiR378 ACUGGACUUGGAGUCAGAAGGC
	btamiR378* CUCCUGACUCCAGGUCCUGUGU
sGFP	sGFP AGAACGGCAUCAAAGCCAACU
	asGFP UUGGCUUUGAUGCCGUUCUUUU

Synthetized both methylated at the 3′-terminus or not, according to their plant origin and the experiment performed.
