# Supplementary material for: Plant microRNAs as novel immunomodulatory agents
Source: Sci Rep. 2016 May 11;6:25761. doi: 10.1038/srep25761 (PMC4863160; doi:10.1038/srep25761)
Supplement: Supplementary Information [file srep25761-s1.doc]

**Supplementary File**

**Plant miRNAs as novel immunomodulatory agents**

Duccio Cavalieri^1,2,*^, Lisa Rizzetto^1^, Noemi Tocci^1^, Damariz Rivero^3^, Elisa Asquini^1^, Azeddine Si-Ammour^1^, Elena Bonechi^3^, Clara Ballerini^3^, Roberto Viola^1,*^

^1^Research and Innovation Centre, Fondazione Edmund Mach, via E. Mach 1, 38010 San Michele all’Adige (TN), Italy;

^2^Department of Biology, University of Florence, Via Madonna del Piano 6, 50019 Sesto Fiorentino (FI), Italy;

^3^Dipartimento di Neuroscienze, Psicologia, Area del Farmaco e Salute del Bambino (Neurofarba), University of Florence, viale Pieraccini 6, 50139 Firenze, Italy.

* Corresponding authors


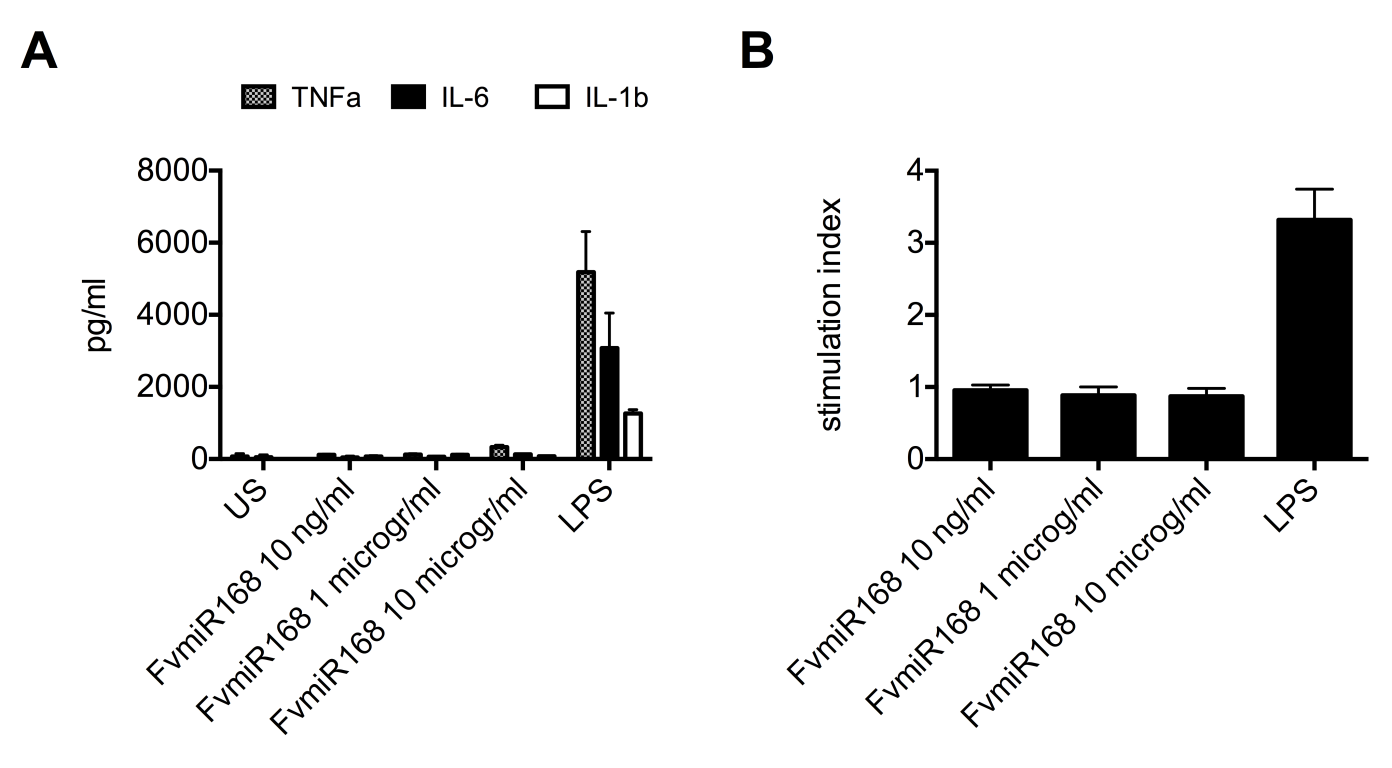


**Supplementary Figure 1.** Effects of plant miRNA treatment in human immune function.

(**a**) Effects of miRNA treatment on DC activation. DCs were exposed to miRNAs (10 ng/ml, 1 µg/ml, 10 µg/ml). After 24 hr IL-1β, TNFα, IL-6 released on culture supernatant. (**b**) Effects of treated DCs in stimulating T cells. T cells were exposed to treated DCs. After five days, proliferation has been measured as [^3^H]-Thy uptake by liquid scintillation [^3^H]-Thy. MLR results are shown. Proliferation is presented as stimulation index, percentage stimulation above background is determined for each stimulated sample, through comparison with results from an unstimulated sample. In both experiments, LPS has been used as positive control. Mean ± SD, N=3, *p<0.05, **p<0.01, Student t-test, treatment vs no treatment.


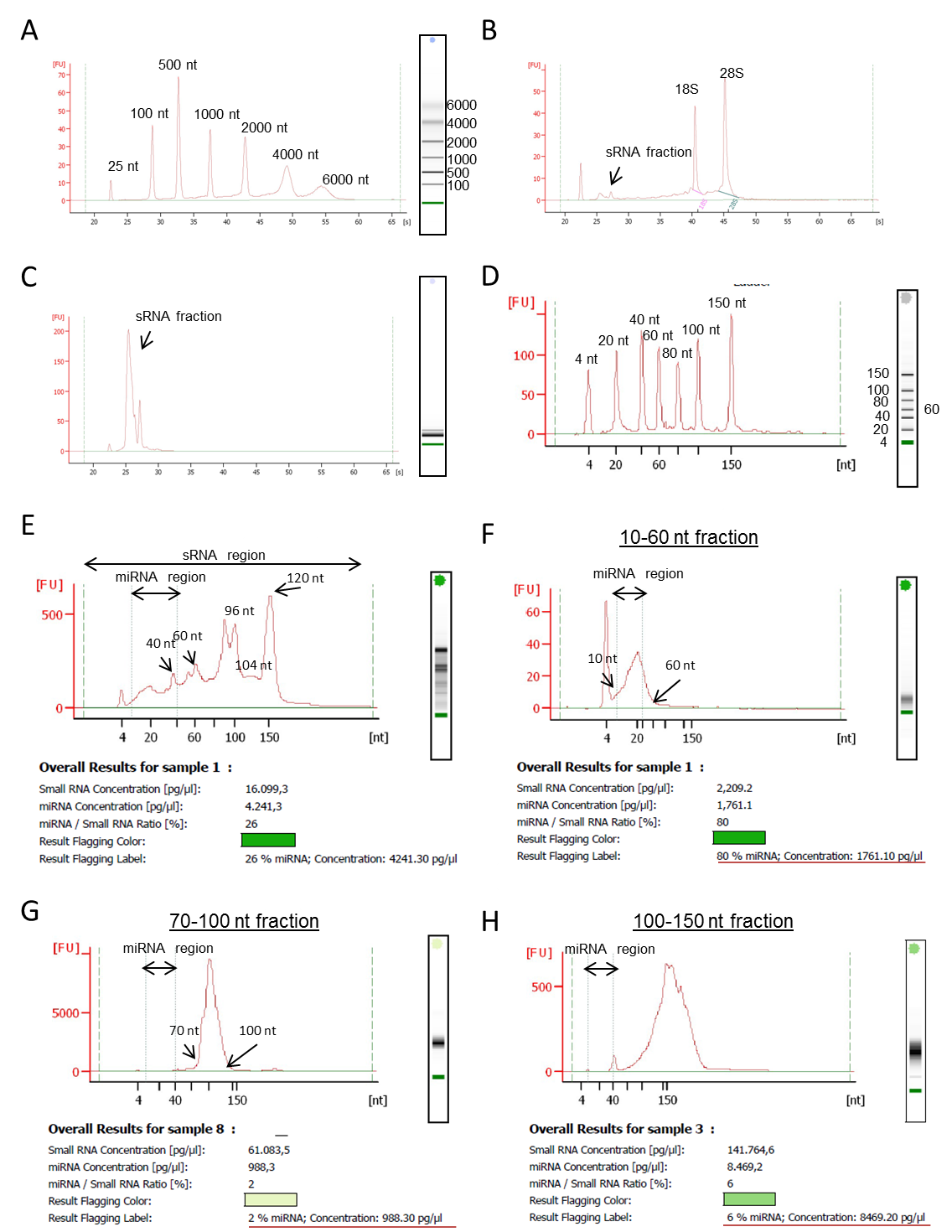


**Supplementary Figure 2.** Examples of the sRNA profiles obtained according to Material and Methods.

(**a**) Ladder profile showing the six large RNA markers in nucleotides (nt), according to RNA 6000 Nano kit (Agilent); (**b**) Profile obtained by running a total plant RNA sample encompassing the high molecular weight fraction, identifiable by the 28S and 18S peaks, and the sRNA fraction of low molecular weight, on the left of the electropherogram. (**c**) Example of a plant total sRNA extracted with the Cetrimonium bromide (CTAB) protocol and used in the experiments presented, in particular cabbage leaves sRNA in the figure; (**d**) Ladder profile showing the six small RNA markers in nucleotides (nt), according to small RNA Nano kit (Agilent); (**e**) Profile obtained by running a total small RNA sample, extracted according to CTAB extraction protocol. (**f-g-h**) Example of the sRNA fractions obtained by gel fractioning of total sRNA and after the quality check used in the experiments presented.


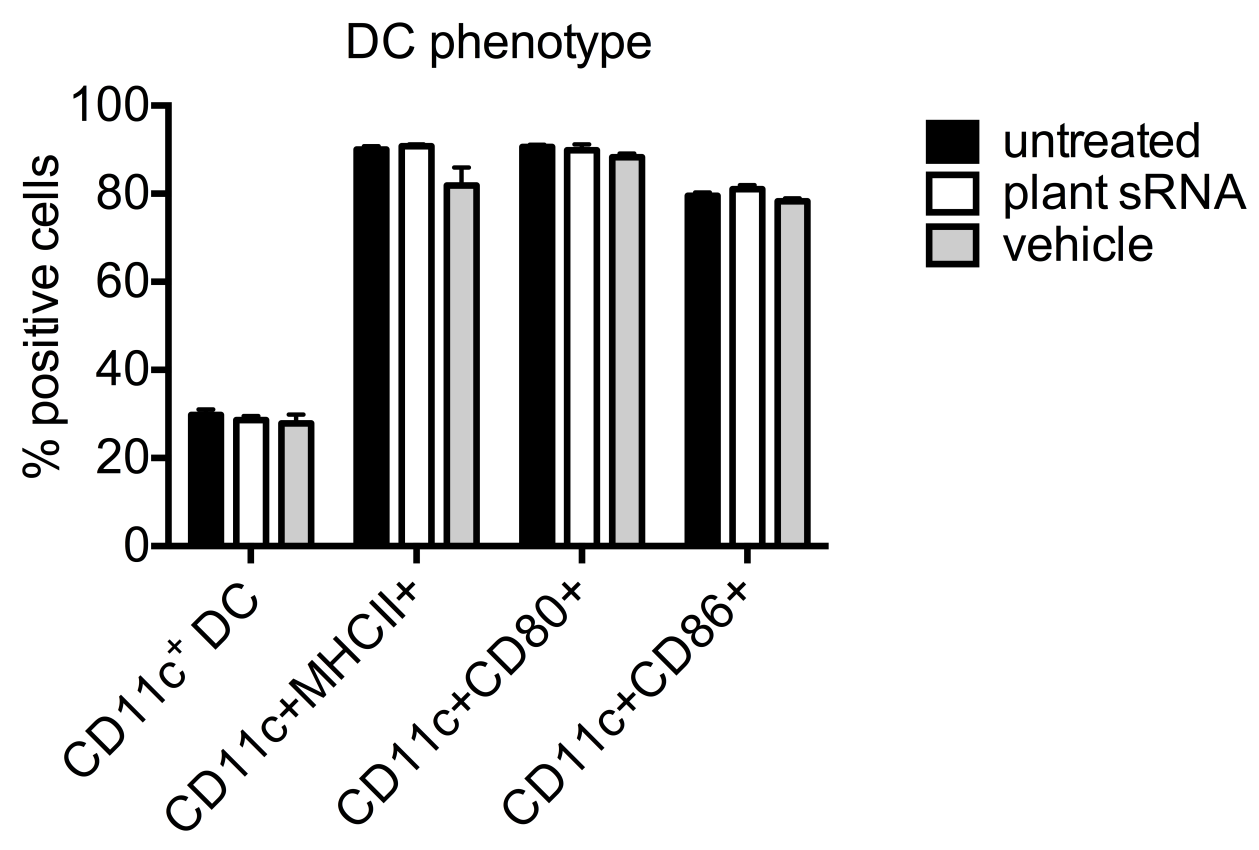


**Supplementary Figure 3.** Effects of plant miRNA treatment on mouse lymphnode isolated DCs.

To characterize the DCs population present in the samples, lymphnode CD11c^+^ DCs were isolated from draining lymphonodes of all the experimental groups and analyzed by flow cytometry. Levels of MHCII, CD80, CD83 and CD86 expression in treated and untreated animals were measured. Mean ± SD, N=6.

*
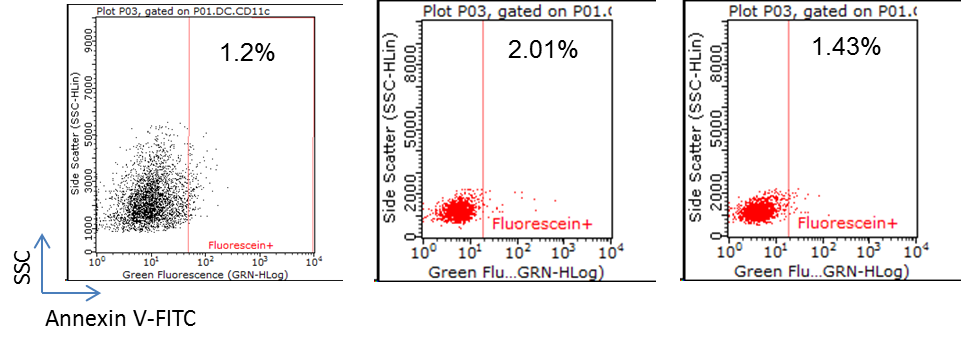
*

**Supplementary Figure 4.** Viability assessment by flow cytometry of human DCs population used in *in vitro* experiments, prior and after experimental readouts.

Representative plots of Annexin V staining of CD11c^+^ gated DCs cells. Annexin V-negative cells, on the left of the plot are representative of viable DCs cells. The percentage indicated on the plots, represents the number of dying cells.
